# Heart Rate Variability and Firstbeat Method for Detecting Sleep Stages in Healthy Young Adults: Feasibility Study

**DOI:** 10.2196/24704

**Published:** 2021-02-03

**Authors:** Liisa Kuula, Anu-Katriina Pesonen

**Affiliations:** 1 SleepWell Research Program University of Helsinki Helsinki Finland

**Keywords:** electroencephalogram, actigraphy, polysomnography, sleep, heart rate, rapid eye movements

## Abstract

**Background:**

Polysomnography (PSG) is considered the only reliable way to distinguish between different sleep stages. Wearable devices provide objective markers of sleep; however, these devices often rely only on accelerometer data, which do not enable reliable sleep stage detection. The alteration between sleep stages correlates with changes in physiological measures such as heart rate variability (HRV). Utilizing HRV measures may thus increase accuracy in wearable algorithms.

**Objective:**

We examined the validity of the Firstbeat sleep analysis method, which is based on HRV and accelerometer measurements. The Firstbeat method was compared against PSG in a sample of healthy adults. Our aim was to evaluate how well Firstbeat distinguishes sleep stages, and which stages are most accurately detected with this method.

**Methods:**

Twenty healthy adults (mean age 24.5 years, SD 3.5, range 20-37 years; 50% women) wore a Firstbeat Bodyguard 2 measurement device and a Geneactiv actigraph, along with taking ambulatory SomnoMedics PSG measurements for two consecutive nights, resulting in 40 nights of sleep comparisons. We compared the measures of sleep onset, wake, combined stage 1 and stage 2 (light sleep), stage 3 (slow wave sleep), and rapid eye movement (REM) sleep between Firstbeat and PSG. We calculated the sensitivity, specificity, and accuracy from the 30-second epoch-by-epoch data.

**Results:**

In detecting wake, Firstbeat yielded good specificity (0.77), and excellent sensitivity (0.95) and accuracy (0.93) against PSG. Light sleep was detected with 0.69 specificity, 0.67 sensitivity, and 0.69 accuracy. Slow wave sleep was detected with 0.91 specificity, 0.72 sensitivity, and 0.87 accuracy. REM sleep was detected with 0.92 specificity, 0.60 sensitivity, and 0.84 accuracy. There were two measures that differed significantly between Firstbeat and PSG: Firstbeat underestimated REM sleep (mean 18 minutes, *P*=.03) and overestimated wake time (mean 14 minutes, *P*<.001).

**Conclusions:**

This study supports utilizing HRV alongside an accelerometer as a means for distinguishing sleep from wake and for identifying sleep stages. The Firstbeat method was able to detect light sleep and slow wave sleep with no statistically significant difference to PSG. Firstbeat underestimated REM sleep and overestimated wake time. This study suggests that Firstbeat is a feasible method with sufficient validity to measure nocturnal sleep stage variation.

## Introduction

Sleep stages alternate throughout the typical nighttime sleep period. After the initial sleep onset, nonrapid eye movement (NREM) sleep stages 1 (N1), 2 (N2), and 3 (N3) emerge alongside rapid eye movement (REM) sleep [1]. Together, both NREM and REM sleep stages form sleep cycles, which, in healthy adults, rotate approximately four or five times over the course of a single night [2]. This alteration between stages correlates with changes in physiological measures such as muscle tonus [3,4], blood pressure [5-7], temperature regulation [8,9], as well as heart rate and heart rate variability (HRV) [10,11].

More specifically, NREM sleep stages are related to stability in the cardiovascular system and stronger parasympathetic cardiac modulation. This, in turn, is reflected in REM sleep so that the heart rate increases and becomes less stable [5,7]. Within NREM sleep stages, the differences between deep sleep (N3, or slow wave sleep [SWS]) and lighter sleep stages (N1 and N2) also have some physiological differences, but these are less pronounced than those between REM and NREM [12]. Specifically, the deeper the sleep, the stronger the parasympathetic cardiac modulation (ie, SWS is associated with a lower heart rate compared to N2 and N1) [13,14].

Polysomnography (PSG) is considered the gold-standard means for measuring sleep stages, as the combination of electromyography (EMG) and electroencephalograph (EEG) is, by definition, the only way to distinguish between the different sleep stages [1]. Although PSG provides reliable data on sleep, other less laborious methods are needed as the increasing prevalence of sleep disorder diagnoses [15] has highlighted an urgent gap to be filled in the development of reliable, cost-efficient sleep study tools for both clinical and consumer use [16].

Some recent studies suggest that HRV might provide a noninvasive marker for detecting sleep behavior such as differentiating between sleep stages [10,11]. HRV has also been widely utilized for assessing phenomena such as stress and recovery [17], physical activity [18], and oxygen consumption [19]. Recently, a sleep analysis method was developed based on HRV and acceleration data (Firstbeat Technologies Oy, Jyväskylä, Finland) for providing personalized feedback and guidance regarding the quantity and quality of sleep. HRV as measured by a single-lead ECG device (Firstbeat Bodyguard 2, Firstbeat Technologies Oy) can estimate atrial fibrillation accurately [20], making it a reliable measurement device regarding HRV-related phenomena.

Based on the need to evaluate the validity of commonly available and easy-to-administer sleep measurement solutions, we investigated how PSG and the Firstbeat sleep analysis algorithm correlate in detecting sleep stages. From analog measurements, we estimated the sensitivity, specificity, and accuracy of the Firstbeat method in relation to PSG measurement over two nights.

## Methods

### Participants

The study protocol has been described in detail in a previous publication [21]. We recruited 20 voluntary participants (mean age 24.5 years, SD 3.5, range 20-37 years; 10 [50%] women) by word of mouth in Helsinki, Finland. Participants were recruited from the research team’s circle of acquaintances: if the acquaintance showed initial interest in participating, they received a detailed description of the procedure via email. After reading the description, if the potential participant was still interested in taking part in the sleep study, they were screened for suitability. Their sleep was then measured for two consecutive nights using PSG, chest-worn Firstbeat Bodyguard 2, and a wrist-worn Geneactiv actigraph (Activinsights Ltd, Kimbolton, United Kingdom). The inclusion criteria were as follows: aged between 20 and 45 years, and a relatively stable sleep schedule (eg, no shift work or jet lag). Exclusion criteria were any diagnosed sleep disorder, the use of any medication that could affect sleep, acute sickness (eg, the flu), and gold allergy (as the electrodes used for the PSG recording were gold-plated). Each participant received a compensation of 100 euros (US $115) and structured feedback on their sleep stages. Written informed consent was obtained from all participants. The study was approved by the Ethical Committee of the Helsinki University Central Hospital. All procedures followed were in accordance with the Helsinki Declaration and its later amendments.

In our previous study, we investigated two different intervention groups within this setting, and demonstrated that these groups did not differ significantly from each other [21]. The Pittsburgh Sleep Quality Index (PSQI) scores of the participants, ranging from 2 to 12 points, indicate some variation in sleep quality. For the purpose of this study, all nights from all participants were pooled together for comparisons of PSG and Firstbeat sleep metrics.

### Procedure

A research assistant visited participants at their homes on two consecutive nights. Participants had been asked not to consume alcohol or caffeine after 4 PM on the measurement nights. The evening visit started between 6 and 10 PM, depending on the participant’s current sleeping schedule. The research assistant attached the measurement electrodes to the participant during the house call and began the recording. Before the research assistant left, participants were given instructions for the night; the participants were instructed to spend the evening as usual but to refrain from vigorous activities. They were also asked to keep their phones and any other electrical devices with transmitters at least 2 meters away from the bed so they would not interfere with the PSG recording. Participants were instructed to sleep normally, and the visit for the following morning was scheduled according to the participant’s expected wake-up time. The research assistant arrived the following morning approximately 0 to 30 minutes after the wake-up time.

### Physical Measurements

We measured HRV with Firstbeat Bodyguard 2, including two chest electrodes and 3-axis acceleration data obtained from the wrist with a Geneactiv actigraph. The Firstbeat sleep analysis method evaluates the physiological state of the person as being awake or asleep based on HRV and acceleration data, and scores sleep as N1+N2 (light), N3, or REM. The method uses a neural network–based algorithm with HRV, HRV-derived respiration rate, movement, and time of day data for sleep and wake detection and for sleep classification. To align the measurement modes, we combined PSG-measured sleep stages N1 and N2 to correspond to “light” sleep of the Firstbeat method.

We used overnight PSG to measure sleep at home (SOMNOscreen plus, SOMNOmedics GmbH, Germany) with the following recorded parameters: EEG (left and right for F, C, O), left and right electrooculogram (EOG), left and right EMG, and ECG. The setup for the PSG and the removal in the morning were carried out by a trained research assistant. EEG measurements were recorded with gold cup electrodes at 6 EEG locations (F3, F4, C3, C4, O1, and O2) and 2 channels for the mastoids (A1, A2) according to the standardized 10/20 system. The ECG, EOG, and EMG were measured using disposable adhesive electrodes (Ambu Cardiology Blue Sensor M; Ambu Neuroline 715, Ambu A/S, Denmark) with two locations for ECG and EOG, and three locations for EMG. In addition, an online reference Cz and a ground electrode in the middle of the forehead were used. The sampling rate was 256 Hz. All signals were filtered with a pass band of 0.5-40 Hz (Hamming windowed sinc zero-phase FIR filter, cutoff [−6 dB] 0.25 Hz and 44.3 Hz, respectively) and rereferenced to the average signal of A1 and A2 electrodes. Sleep stages from PSG data were scored manually with the DOMINO program version 2.7 (SOMNOmedics GmbH, Germany) by two experienced researchers in 30-second epochs. The scoring was completed with both researchers visually inspecting the data together and agreeing over each epoch. The scoring was paused if any disagreement emerged and continued after agreement was found based on careful inspection of all channels, in accordance with the rules published by the American Academy of Sleep Medicine (AASM) [1].

### Statistical Analyses

Following standard sleep score practices in the AASM manual [1], we used 30-second epochs for sleep stage comparisons. The entire data were compared side by side after lights off; following AASM scoring rules, sleep onset was defined as the first epoch of any sleep stage as detected by the PSG measurement. We compared how Firstbeat was able to detect the actual sleep onset by calculating the difference between the two time points, which were statistically evaluated using a paired *t* test. All comparisons of sleep staging between PSG and Firstbeat were performed from the PSG-measured actual sleep onset onward.

First, we used paired-sample *t* tests to compare sleep metrics for evaluating differences between PSG and Firstbeat in sleep onset, and minutes spent in wake, light sleep, SWS, and REM sleep. Second, we conducted epoch-by-epoch comparisons between Firstbeat and PSG to calculate the sensitivity (ability of Firstbeat to detect true sleep), specificity (ability of Firstbeat to detect true wake), and accuracy (ability of Firstbeat to detect both sleep and wake). This comparison was performed across all sleep stages, as well as for overall sleep-wake comparisons between PSG and Firstbeat.

Third, we used a confusion matrix to compare epoch-by-epoch measures of true positives, true negatives, false positives, and false negatives between Firstbeat and PSG across all measured nights for sleep versus wake as well as for light sleep, SWS, and REM sleep stages. True positives arise when both the PSG and Firstbeat score the 30-second epoch as sleep. True negatives arise when both the PSG and Firstbeat score the epoch as awake. False positives arise when the PSG scores the epoch as sleep but Firstbeat scores it as wake. False negatives arise when the PSG scores the epoch as wake but Firstbeat scores it as sleep.

Fourth, we evaluated the differences between the amount of sleep scored as wake, light sleep, SWS, or REM sleep when comparing Firstbeat and PSG using minute-based Bland-Altman plots, and visually demonstrate how many observations remained within a 30-minute window of the PSG measure.

Finally, we used *t* tests to compare whether specificity, sensitivity, and accuracy differed based on sex, measurement night, or the intervention we reported previously [21].

## Results

The 40 nights from 20 participants measured with both PSG and Firstbeat were included in all analyses with no exclusions. Table 1 shows the participants’ characteristics as well as their mean sleep measures.

**Table 1:** Characteristics of the sample (N=20).

Characteristic			Value
Age (years), mean (SD)			24.50 (3.50)
Sex (female), n (%)			10 (50)
BMI (kg/m^2^), mean (SD)			23.64 (3.10)
PSQI^a^ score, mean (SD)			5.40 (2.35)
Poor sleep quality (PSQI score>5), n (%)			5 (25)
**Polysomnography-measured sleep, mean (SD)**			
	Sleep onset (hour:minute)		23:44 (1:05)
	TST^b^ (hour:minute)		7:28 (0:49)
	Sleep efficiency (%)		91.55 (5.82)
	N1^c^ of TST (%)		4.68 (2.71)
	N2^d^ of TST (%)		47.46 (6.14)
	N3^e^ of TST (%)		22.18 (7.11)
	REM^f^ of TST (%)		25.68 (5.48)

^a^PSQI: Pittsburgh Sleep Quality Index.

^b^TST: total sleep time.

^c^N1: sleep stage 1 (light sleep).

^d^N2: sleep stage 2 (light sleep).

^e^N3: sleep stage 3 (slow wave sleep).

^f^REM: rapid eye movement.

Table 2 shows paired *t* test comparisons and the mean differences between Firstbeat and PSG sleep stage scores. Sleep onset did not differ significantly between Firstbeat and PSG (mean difference 0, SD 9 minutes, SE 1 minute; *t*_39_=0.578, *P*=.57). Three nights’ sleep onset was detected accurately, whereas for 12 nights, the Firstbeat method assumed earlier sleep onset than PSG. To detect the true difference in detecting sleep onset, we calculated the absolute difference between Firstbeat onset to PSG onset, and found a mean difference of 7.06 minutes (SD 6.64 minutes).

**Table 2:** Paired comparisons and mean differences of sleep parameters recorded by Firstbeat and polysomnography.

Parameter (minutes)	Mean difference^a^ (SD)	SE	95% CI	*t* (*df*=39)	*P* value
Wake	14.03 (16.65)	2.63	8.70 to 19.35	5.327	<.001
Light sleep	–0.80 (42.25)	6.68	–14.31 to 12.72	–0.120	.91
Slow wave sleep	4.68 (46.79)	7.40	–10.29 to 19.64	0.632	.53
REM^b^ sleep	–17.90 (50.44)	7.98	–34.03 to –1.77	–2.244	.03

^a^Mean differences calculated as Firstbeat – polysomnography.

^b^REM: rapid eye movement.

When comparing Firstbeat and PSG, there were some differences in how well the Firstbeat method was able to detect different sleep stages. The mean specificity, sensitivity, and accuracy in detecting wake was 0.77 (SD 0.16), 0.95 (SD 0.03), and 0.93 (SD 0.03), respectively. The specificity, sensitivity, and accuracy in detecting light sleep was 0.69 (SD 0.15), 0.66 (SD 0.10), and 0.69 (SD 0.06), respectively. The specificity, sensitivity, and accuracy in detecting SWS was 0.91 (SD 0.06), 0.72 (SD 0.17), and 0.87 (SD 0.04), respectively. REM sleep was detected with 0.92 (SD 0.7) specificity, 0.60 (SD 0.24) sensitivity, and 0.84 (SD 0.06) accuracy.

Table 3 shows the confusion matrix [22] regarding the two measurement devices and their differences.

**Table 3:** Confusion matrix of the Firstbeat method and polysomnography sleep stage epoch comparisons.

Firstbeat (N)	Polysomnography (N)				
	Light	SWS^a^	REM^b^	Wake	Total
Light	12,737^c^	2189	3445	492	18,863
SWS	2683	5867^c^	40	96	8686
REM	2072	131	5576^c^	205	7984
Wake	1435	125	355	2598^c^	4513
Total	18,927	8312	9416	3391	40,046^c^
Correct stage classification (%)	67.3	70.6	59.2	76.6	N/A^d^

^a^SWS: slow wave sleep.

^b^REM: rapid eye movement.

^c^Diagonals indicate the number of correctly categorized epochs in the respective sleep stage.

^d^N/A: not applicable.

**Figure 1:**
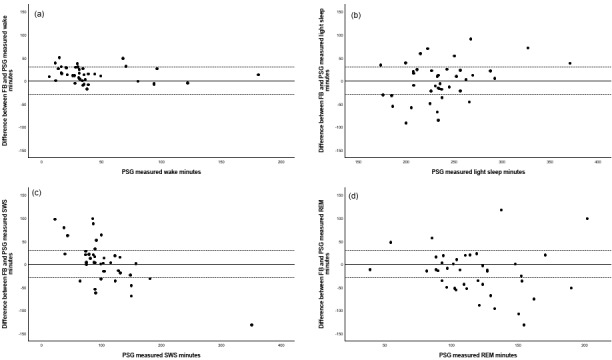
Bland-Altman plots comparing differences in Firstbeat (FB) and polysomnography (PSG) in wake state (a), light sleep stages N1+N2 (b), slow wave sleep (SWS) (c), and rapid eye movement (REM) sleep (d).

Figure 1 shows the Bland-Altman mean difference plots, which illustrate the share of observations that were within 30 minutes from each other in wake state, or in different sleep stages as measured with different devices.

As a further sensitivity check, we compared the means of specificity, sensitivity, and accuracy to detect possible differences based on sex, measurement night, or the intervention reported previously [21]. Compared to females, there was a better specificity in REM sleep in male participants (0.95 vs 0.89, *P*=.004), but there were no other differences between sexes (*P*>.06). There was no first- or second-night effect in the specificity, sensitivity, and accuracy (all *P*>.38), nor regarding the presence of the previously reported music or slow-breathing intervention (all *P*>.13).

## Discussion

### Principal Findings

Wearable devices have gained a significant share of the health and well-being consumer market, and new wearable devices and algorithms emerge frequently. Although a great majority of this research aims to detect sleep quality and duration based on data derived from accelerometer sensors [23], other measures such as respiratory signals have also been utilized [24]. Several reviews have evaluated the accuracy of accelerometer-based sleep wearables [23,25,26], and a recent review summarized an overall evaluation of wearables utilizing other sensors [27]. They concluded that detecting sleep from wake is relatively successful in many devices, but when wearables aim to classify sleep stages as opposed to simply distinguish between sleep and wake, there is a challenge in distinguishing four choices (wake, light, deep, and REM sleep) [27], which makes the result more inaccurate.

Commercial accelerometers typically yield accuracy between 0.81 and 0.91, sensitivity values between 0.87 and 0.99, and specificity values between 0.10 and 0.52 in distinguishing sleep from wake [26]. However, when attempting to detect sleep stages, the results are less consistent. A recent review focusing on commercial accelerometers identifying sleep stages found great variation in accuracy depending on the study [26]. For instance, accuracy in detecting light sleep varied between 69% and 81%, accuracy in detecting SWS was between 36% and 89%, and that for REM sleep ranged between 62% and 89%. Such variation suggests that acceleration itself may not be sufficient in reliably identifying sleep stages.

Previous studies have implied that HRV may be a useful marker for detecting sleep stages [10,11]. One study reported an accuracy of up to 89% in detecting SWS, but their method included respiratory signals alongside HRV [28]. When detecting sleep stages by utilizing both HRV and accelerometer data, one study managed to identify 75% of SWS correctly [29]. In that study, REM sleep was identified correctly in over 70% of epochs, whereas light sleep detection was the weakest with correct identification varying between 42% and 52%. Our findings are of similar accuracy, which further supports the notion of combining accelerometer and HRV-based measures for reproducible sleep staging.

This study was performed to evaluate the ability of HRV- and acceleration-based Firstbeat sleep analysis methods to detect sleep and different sleep stages. In pairwise comparisons, the Firstbeat method detected light sleep and SWS with no statistically significant difference to the gold-standard PSG method. There were two measures that differed significantly between the Firstbeat method and PSG: Firstbeat underestimated REM sleep (mean 18 minutes) and overestimated wake (mean 14 minutes). Considering the number of minutes in the context of a typical night’s sleep, the differences are not alarmingly high in practice, especially when measuring sleep over repeated nights. Sleep onset detection was very accurate, which is in accordance with a review published earlier this year [30].

Sleep stages can only be detected using PSG, as the stages are, by definition, separated by different patterns in ECG, EOG, and ECM. REM sleep is particularly difficult to detect without measuring activity from EOG and EMG channels. Thus, relying on other physiological measures as a means for separating sleep stages is always based on secondhand information. Although HRV has both previously [10,11] and in this study reflected sleep stages relatively well, it cannot detect the immediate changes in EEG, EOG, and EMG. However, this study suggests that HRV-assisted sleep stage detection can serve as a good estimate of sleep architecture despite being less accurate in detecting specific sleep stages.

When observing the comparisons in more analytical detail, we found that comparing the Firstbeat method against PSG yielded good specificity, and excellent sensitivity and accuracy in detecting wake. Regarding light sleep, the measures of specificity, sensitivity, and accuracy were less convincing. SWS detection had excellent specificity, adequate sensitivity, and good accuracy, while REM sleep was detected with similarly excellent specificity, adequate sensitivity, and good accuracy. These results suggest that the Firstbeat method is best at detecting sleep stages that have strong parasympathetic cardiac markers; however, light sleep is typically not significantly differentiated based its physiological fingerprint [12,14].

### Strengths and Limitations

Our study was fully balanced in sex distribution and we were able to evaluate the Firstbeat method across two different nights in two different settings in the participants’ own homes. Thus, the ecological validity in this study can be considered excellent.

As a limitation, even though our sample had some variation in PSQI-measured sleep quality, this study did not include any participants with diagnosed sleep disorders. Our study included only healthy participants, and the results are likely to be different if any health issues, particularly cardiovascular, or any sleep disorders are present. This is a question to solve before utilizing the Firstbeat method in clinical contexts.

### Conclusion

Combining HRV with accelerometer measurements can be considered a feasible method with sufficient validity to measure nocturnal sleep stage variation. We found that the specificity, sensitivity, and accuracy were the weakest in detecting light sleep. Nevertheless, considering its availability, affordability, and ease of administration, Firstbeat may be a useful tool in various contexts, particularly in consumer-based sleep-measuring environments to produce an overview of sleep structures.

## Abbreviations

AASM: American Academy of Sleep Medicine
EEG: electroencephalography
EMG: electromyography
EOG: electrooculogram
HRV: heart rate variation
N1: stage 1 (nonrapid eye movement) sleep
N2: stage 2 (nonrapid eye movement) sleep
N3: stage 3 (nonrapid eye movement) sleep (slow wave sleep)
NREM: nonrapid eye movement
PSG: polysomnography
PSQI: Pittsburgh Sleep Quality Index
REM: rapid eye movement
SWS: slow wave sleep
